# Annotations

**Published:** 1888-02-18

**Authors:** 


### Annotations.

**A New Occupation for Women.**

Two young women, it is said, are farming on their own account at Auburndale, near Boston, and are obtaining highly profitable results. They were not, it appears, bred to farming, but to school teaching, which they gave up two or three years ago to try their hands at agriculture. They began in a very elementary way by raising chickens, and now their fowls and eggs are famous all the country round. Each egg is separately dated on the day it is laid, and thus, so long as the ladies are honest, buyers have an absolute guarantee of the freshness of their eggs. For this guarantee many people are willing to pay twice the price of ordinary eggs. With true business instinct the ladies take care that everything they produce is of the best; and, what is almost as important, that it is sent to market in the most presentable shape. Their results are not produced without hard work; and sometimes, though probably not often, they have to rise as early as three o'clock in the morning. This little story is well worthy of much meditation on the part of the ladies of this country. Farming is by no means a difficult business to learn, especially hen-farming, fruit-farming, and other such branches of the agricultural art. These departments are eminently suitable occupations for women, and particularly for delicate women, being mostly carried on in the open air. They demand a certain degree of gentleness, neat-handedness, and care in detail—exactly the kind of attention women are best fitted to give. The experiment tried by the American ladies is by no means unknown in England. Many a farmer's wife who is skilful in the dairy and in the henyard saves her husband in these hard times from the tender mercies of the money-lender. If we, as a people, were but a little more imaginative and a little more practical at the same time, how many intelligent young women might soon be put into the way of making a good living and establishing a healthy constitution by being each placed on four or five acres of land! The land is here, and is going rapidly out of cultivation; the women are here, and are destroying each other by over-competition in every market to which they have access; the capital is here, and is so plentiful, that it is hardly worth lending out at the rate of interest obtainable. What hinders the making of a few inexpensive experiments by lady farmers? Nothing but the want of imagination, courage, and sense.

**The Morals of the Charitable.**

Miss Louisa Twining objects to bazaars in aid of charitable objects, and not without good reason Most persons who give money in charity have a certain regard for morality, and some of them possess an elementary knowledge of business. Those who have had experience of church, hospital, and other bazaars, know very well that all kinds of practices are resorted to which, tried by any recognized standard of morals, cannot be justified. Gambling is believed by the average Englishman to be a cardinal sin—a practice deserving of the severest reprobation. Yet at most bazaars gambling is rampant under the very nose of the men and matrons who in the abstract regard it with horror and detestation. What can be the reason of this? Has the average Englishman so poor an intellect that he cannot detect a thing in the concrete which he understands and hates in the abstract? Or does he think that the end sanctifies the means? This last is a most dangerous doctrine, and often leads people, who have beforetime been blameless, into quagmires of wickedness from which they never escape again. But apart from the immorality to which bazaars almost invariably give rise, the business aspect of the case deserves to be soberly considered. At a bazaar recently held on behalf of a certain hospital, the gross receipts amounted to close upon nineteen hundred pounds. Of this sum nearly seven hundred disappeared in expenses, leaving little more than twelve hundred for the hospital funds. It is practically certain that the real value of the articles sold amounted to two thousand four hundred pounds. In that case, therefore, the people who provided the goods realized less than the sum actually paid for the raw materials; and all the labour of manufacture, together with the almost indescribable worry connected with removal to the place of sale, and the actual selling, were worse than thrown away. If the ladies who take bazaars in hand would give in money what they themselves can afford, and take contributions also in money from their friends—male and female—instead of in manufactured articles, a vast amount of useless labour would be spared, much very doubtful morality would be avoided, and charitable objects would in most instances gain largely by the change. Of course cynical people will pay little heed to the moral aspect of the question, but we venture to think that cynical people do not generally waste much money in public or private charity. We urge upon our readers the necessity of giving to the subject some serious consideration.

**What Dreams are Made Of.**

"We are such stuff as dreams are made of," said Shakespeare; and having the good fortune to live in the sixteenth century, and not in the nineteenth, he could make his little reflection without any cruel scientist coming up to ask him, "And what are our dreams made of, sir? Answer me that." Shakespeare could not have answered. M. Max Simon makes a courageous attempt to do so. His conclusion is that the phenomena and causes of dreams are much akin to those of madness and hallucinations. In dreams the wayward fancy has things all its own way, uncontrolled by reason, and makes the most of it, founding on some fragment of physical fact a whole edifice of airy fiction, which, however, is in some cases not far removed from the truth. This is often the case where the dreams predict disease. M. Teste, one of Louis Philippe's Ministers, dreamed that he had an attack of apoplexy, and such an attack came on within three days and killed him. Galen had a patient who dreamed he had "a leg of stone," and who soon afterwards suffered from paralysis of the leg. It is evident that with these dreamers a physical uneasiness, premonitory of disease, caused a fanciful interpretation of the symptoms, and this forms the explanation of most of the delirium of fever. But M. Simon believes that many dreams are suggested by such trivial means as unconscious puns, and tells the history of a long and complicated dream of his own, arising from such a cause. If so much can spring from one small pun, it is too awful to think of the nightly visions of an inveterate and confirmed punster. What a phantasmagoria these must suffer from! An intellectual indigestion! The only people who, in these days—when prophesy is at a discount, and the "signs of the times" take the form of a newspaper sold at all bookstalls for a penny—make any profit out of dreams are the bookmakers, those of the turf and those of Paternoster Row. Perhaps the profit of the first of these is small. They back horses on the strength of dreams, and lose by the experiment oftener than they win. The others, the literary bookmakers, benefit more. Mr. Louis Stevenson tells how the central idea of "Dr. Jekyll and Mr. Hyde" was brought to him by "his Brownies" in a dream; and Coleridge blamed the interruption of a dream for the unfinished condition of "Kubla Khan." It is true that Coleridge's genius, great as it was, was less than his laziness; and that though Mr. Stevenson's imagination never runs away with him, this is due to his having an exceptionally firm seat on his Pegasus, not to its shirking the boldest flights. Perhaps the dreams had less to do with the finished story and the unfinished poem than their authors pretend; but if these be dreams, why do not such brilliant ones disturb the slumbers of us duller folk!

